# Identifying new targets for rectal cancer treatment

**DOI:** 10.18632/oncoscience.508

**Published:** 2020-05-14

**Authors:** Sylvain Ferrandon, Matthew F. Kalady

**Affiliations:** ^1^Cancer Biology Department, Lerner Research Institute, Cleveland Clinic, Cleveland, Ohio, USA; ^2^Department of Colorectal Surgery, Digestive Disease and Surgery Institute, Cleveland Clinic, Cleveland, Ohio, USA

**Keywords:** COASY, rectal cancer, radiation resistance

Colorectal cancer remains one of the highest incident and top causes of cancer-related mortality in the United States and around the world. In addition to oncologic outcomes, rectal cancer in particular presents additional challenges as surgery for rectal cancer can cause significant decreases in bowel function and quality of life, or even result in a permanent colostomy. The adoption of multimodality care with radiation and chemotherapy before surgery has improved cancer outcomes. In fact, response to neoadjuvant chemoradiation is directly correlated with survival [1]. Furthermore, a subset of patients with an excellent clinical response to chemoradiation may avoid surgery and preserve function [2]. Unfortunately, there is great variety among patients in their response to treatment, and radiation resistance remains a major challenge to successful rectal cancer care [3]. Identifying reasons for this heterogeneity in response, and discovering novel targets to improve radiation sensitivity will lead to treatment advances. We have recently discovered Coenzyme A synthase (COASY) as a novel protein that serves both as a predictive marker for, and has a critical function in radiation resistance [4].

To evaluate patient heterogeneity, we analyzed *COASY* expression in pretreatment human rectal cancers and found levels varied among patients, and that increased levels were directly related to radiation resistance. Using genetic manipulation with *COASY* knockdown and overexpression lines, this observation was confirmed in *in vitro* and *in vivo* models. Mechanistically, we demonstrated a direct interaction between COASY protein and the PI3K regulatory subunit PI3K-P85α, which increased AKT and mTOR phosphorylation, enhancing cell survival after irradiation. Additionally, shRNA *COASY*-knockdown increased DNA double-strand breaks after irradiation [4].

COASY was first described as the mitochondrial enzyme that catalyzes the two last steps of Coenzyme A synthesis in humans. The first reaction, mediated by the PPAT (4′phosphopantetheine adenylyltransferase) domain, is a transfer of the adenosine monophosphate moiety of ATP to 4′-phosphopantetheine to form dephospho-CoA in an Mg^2+^ dependent manner. The last step in CoA synthesis is catalyzed by the dephospho-CoA kinase (DPCK) domain of COASY that adds a phosphate group from ATP to the 3′-hydroxyl of dephospho-CoA [5]. However, the newly discovered non-canonical functions of COASY have broadened its implications in cell survival. COASY has been implicated in the maintenance of DNA integrity and mitotic fidelity in different models such as zebrafish and human breast cancer cells [6, 7]. Recently, a hot spot mutation in the *COASY* gene was observed in a subset of neurodegeneration patients with brain iron accumulation (NBIA) and a distinct set of symptoms, accordingly named COASY protein-associated neurodegeneration (CoPAN). These non-canonical functions of COASY are suspected to be altered and responsible for the cell death observed in the CoPAN phenotype.

A proline-rich domain has been found in the N-terminal extension of COASY. This 29 amino-acids sequence is responsible for the protein-protein interaction with the SH3 domains of the Src family non-receptor tyrosine kinases, Fyn and Csk [8]. The interaction leads to a global phosphorylation of tyrosine residues present in COASY which is necessary for its interaction with PI3K-P85α to promote the activation of the PI3K pathway [9]. Moreover, other proteins are known to interact with PI3K-P85α and exert a cell protection to radiation by modulating the PI3K complex enzymatic activity, such as Fyn, LYN etc. [10, 11]. Our identification of *COASY* as a key mediator of PI3K/Akt radiation resistance signaling provides additional rational for developing COASY protein-targeted therapeutics.

Our findings may have broader implications beyond rectal cancer as the level of *COASY* was found to be significantly overexpressed in tumors compared to normal tissues in several other cancers [4]. Furthermore, the ubiquitous role of PI3K/Akt signaling in cancer suggests that our results may be relevant to several cancer types. This may play a role in other cancers that have variable responses to radiation. By delineating the underlying mechanisms and a link with PI3K pathway activation, our work sheds light on the potential of COASY as a new therapeutic target for future study as a potential radiation-sensitizing agent.
